# Subthalamic deep brain stimulation for refractory Gilles de la Tourette’s syndrome: clinical outcome and functional connectivity

**DOI:** 10.1007/s00415-022-11266-w

**Published:** 2022-07-21

**Authors:** Lulin Dai, Wenying Xu, Yunhai Song, Peng Huang, Ningfei Li, Barbara Hollunder, Andreas Horn, Yiwen Wu, Chencheng Zhang, Bomin Sun, Dianyou Li

**Affiliations:** 1 Department of Neurosurgery, Center for Functional Neurosurgery, Ruijin Hospital, Shanghai Jiao Tong University School of Medicine, Shanghai, China; 2 Department of Neurosurgery, Shanghai Children’s Medical Center, Affiliated to the Medical School of Shanghai Jiao Tong University, Shanghai, China; 3 Movement Disorders and Neuromodulation Unit, Department of Neurology, Charité - Universitätsmedizin Berlin, Berlin, Germany; 4 Einstein Center for Neurosciences Berlin, Charité - Universitätsmedizin Berlin, Berlin, Germany; 5 Berlin School of Mind and Brain, Humboldt-Universität zu Berlin, Berlin, Germany; 6 Center for Brain Circuit Therapeutics, Department of Neurology, Brigham and Women’s Hospital, Boston, MA USA; 7 MGH Neurosurgery and Center for Neurotechnology and Neurorecovery (CNTR) at MGH Neurology, Massachusetts General Hospital, Boston, MA USA; 8 Department of Neurology, Center for Functional Neurosurgery, Ruijin Hospital, Shanghai Jiao Tong University School of Medicine, Shanghai, China; 9 Shanghai Research Center for Brain Science and Brain-Inspired Technology, Shanghai, China

**Keywords:** Subthalamic nucleus, Deep brain stimulation, Gilles de la Tourette’s syndrome, Psychiatric comorbidity, Clinical outcome, Functional connectivity

## Abstract

**Background:**

Deep brain stimulation (DBS) is a promising novel approach for managing refractory Gilles de la Tourette’s syndrome (GTS). The subthalamic nucleus (STN) is the most common DBS target for treating movement disorders, and smaller case studies have reported the efficacy of bilateral STN-DBS treatment for relieving tic symptoms. However, management of GTS and treatment mechanism of STN-DBS in GTS remain to be elucidated.

**Methods:**

Ten patients undergoing STN-DBS were included. Tics severity was evaluated using the Yale Global Tic Severity Scale. The severities of comorbid psychiatric symptoms of obsessive–compulsive behavior (OCB), attention-deficit/hyperactivity disorder, anxiety, and depression; social and occupational functioning; and quality of life were assessed. Volumes of tissue activated were used as seed points for functional connectivity analysis performed using a control dataset.

**Results:**

The overall tics severity significantly reduced, with 62.9% ± 26.2% and 58.8% ± 27.2% improvements at the 6- and 12-months follow-up, respectively. All three patients with comorbid OCB showed improvement in their OCB symptoms at both the follow-ups. STN-DBS treatment was reasonably well tolerated by the patients with GTS. The most commonly reported side effect was light dysarthria. The stimulation effect of STN-DBS might regulate these symptoms through functional connectivity with the thalamus, pallidum, substantia nigra pars reticulata, putamen, insula, and anterior cingulate cortices.

**Conclusions:**

STN-DBS was associated with symptomatic improvement in severe and refractory GTS without significant adverse events. The STN is a promising DBS target by stimulating both sensorimotor and limbic subregions, and specific brain area doses affect treatment outcomes.

**Supplementary Information:**

The online version contains supplementary material available at 10.1007/s00415-022-11266-w.

## Introduction

Gilles de la Tourette’s syndrome (GTS) is a childhood-onset neuropsychiatric disorder characterized by multiple motor tics and one or more vocal tics [1]. Behavioral and psychopharmacological interventions are implemented for patients with GTS as first- and second-line treatments, respectively [1]. However, a subset of patients do not clinically respond to these treatments. For these refractory cases, deep brain stimulation (DBS) has emerged as a promising novel approach for managing the symptoms of severe and refractory GTS [2].

Various brain areas, including the thalamus, globus pallidus internus (GPi), globus pallidus externus, nucleus accumbens, and anterior limb of the internal capsule, have served as investigational DBS targets to alleviate GTS symptoms [3]. According to the results of the International Tourette Syndrome Deep Brain Stimulation Database and Registry, DBS can result in significant improvements in patients’ tics [4–6], but they failed to identify any significant differences across different targets. Despite this, the effectiveness of the most often used targets (GPi and central median thalamus) varies individually and has limited efficacy for their comorbid psychiatric disorders [7–9]. GPi-DBS combined with ablative procedures (anterior limb of internal capsulotomy or cingulotomy) may be considered for treatment-refractory patients with severe self-injurious behavior [10]. However, these ablative procedures are irreversible.

The subthalamic nucleus (STN) is the most common DBS target for the treatment of movement disorders [11]. The efficacy of STN-DBS on Parkinson’s disease (PD) has been established, and promising results for dystonia in open-label studies have been demonstrated [12, 13]. In 2009, Martinez-Torres et al. reported the first case of STN-DBS for a patient with PD with a history of tics [14], which showed that bilateral STN-DBS treatment might also be effective for relieving tic symptoms. The STN is a core basal ganglia structure involved in the control of motor, cognitive, motivational, and affective functions. These functions are distinct in the sensorimotor, cognitive/associative, and limbic subregions based on the topography of cortical projections [15]. According to a few studies, STN-DBS therapies for Tourette syndrome (TS) focused on the stimulation of dorsal part, also as the sensorimotor STN [14, 16], can result in significant improvements in patients’ tics. Additionally, targeting the associative-limbic STN has been effective for obsessive–compulsive behavior (OCB), the most common psychiatric comorbidity of GTS [17, 18]. Based on these promising studies, the STN might serve as an alternative target to treat GTS; however, currently, the nucleus has received little attention in DBS studies for severe and treatment-refractory GTS.

Several studies have reported a correlation between electrode connectivity and improvement in tic symptoms [19, 20]. In this study, we reported the 1-year follow-up clinical outcomes of the so-far largest cohort of STN-DBS with GTS (*n* = 10). Additionally, a whole-brain map of functional connectivity related to the stimulation effect was derived based on the precise active electrode location to illustrate the mechanism of the efficacy of STN-DBS.

## Materials and methods

### Patient selection

Ten patients with refractory GTS who were considered for neurosurgery at Ruijin Hospital from August 2019 to March 2021 were recruited in this study. All subjects met the criteria and were followed up. Demographic and clinical characteristics of each patient are summarized in Table 1. Written informed consent was obtained from all subjects, and the clinical protocol (Institutional Review Board #2018236) was approved by the Ethics Committee of the Ruijin Hospital, Shanghai Jiao Tong University School of Medicine. Data that had been obtained during routine outcome monitoring were incorporated into the clinical care delivered to patients.

**Table 1:** Patient demographic information and clinical characteristics

No	Sex	Age of onset	Age of surgery	Comorbidities	Medications at time of DBS surgery	Motor tics: body regions involved
1	M	9	20	None	Haloperidol, 8 mg/day; benzhexol hydrochloride, 8 mg/day; topiramate, 100 mg/day	Neck, shoulders, legs, feet
2	M	5	14	None	Aripiprazole, 10 mg/day	Eyes, neck
3	M	12	15	None	Aripiprazole, 10 mg/day; tiapride, 600 mg/day; citicoline, 0.3 g/day	Arms, trunk, legs
4	M	8	38	Anxiety	Risperidone, 1 mg/day	Nose, mouth, neck, arms, hands, legs
5	F	6	18	OCB, depression	Aripiprazole, 15 mg/day	Eyes, nose, mouth, neck, shoulders, arms, hands
6	M	11	38	None	Tiapride, 100 mg/day	Eyes, mouth, neck, shoulders, arms, hands
7	M	12	18	None	Aripiprazole, 7.5 mg/day; benzhexol, 3 mg/day; haloperidol, 9 mg/day	Eyes, mouth, neck, hands, legs
8	M	10	26	OCB	Benzhexol, 2 mg/day; topamax, 30 mg/day; aripiprazole, 5 mg/day	Eyes, nose, shoulders, trunk
9	M	5	30	OCB	Haloperidol, 4 mg/day; fluoxetine, 20 mg/day	Neck, shoulders, arms, legs, feet
10	M	5	14	None	Baclofen, 20 mg/day; topiramate, 100 mg/day	Eyes, mouth, neck

Abbreviations: *DBS* deep brain stimulation, *F* female, *M* male, *OCB* obsessive–compulsive behavior

The inclusion criteria were as follows: (1) a primary diagnosis of GTS according to the Diagnostic and Statistical Manual of Mental Disorders, Fifth Edition (DSM-V) criteria [21]; (2) a score on the Yale Global Tic Severity Scale (YGTSS) > 45 (on a scale from 0 to 100, with lower scores indicating less severe symptoms) [22], with or without the presence of psychiatric comorbidities, including OCB, depression, anxiety, and attention-deficit/hyperactivity disorder (ADHD), leading to significant functional impairment; (3) refractory to commonly used pharmacotherapies (α2 agonists, antipsychotics, and dopamine antagonists) and cognitive behavioral therapy; and (4) informed decision to undergo STN-DBS performed by a multidisciplinary team comprising neurosurgeons, neurologists, and psychiatrists.

The exclusion criteria were as follows: (1) presence of severe self-injury behaviors, a comorbid psychotic disorder, particularly schizophrenia, or a substance use disorder, as assessed by the Mini-International Neuropsychiatric Interview [23]; (2) a cluster A or B personality disorder along with a current diagnosis of a major depressive disorder, assessed according to the DSM-V criteria; (3) presence of secondary and functional tic-like movements (i.e., tics not related to TS); (4) presence of observable brain structural abnormalities on the patient’s magnetic resonance imaging (MRI) scan; and (5) previous participation in a clinical trial.

### Surgical procedure

A day prior to surgery, each patient underwent a 3.0 T MRI examination, and the images were transferred into the Leksell Surgiplan workstation (Elekta, Stockholm, Sweden). On the day of surgery, the Leksell stereotactic head frame was mounted, Helical computed tomography (CT) imaging was performed for image fusion, and the neurosurgeon used the fused images for planning. The initial STN coordinate were 2–3 mm posterior to the midpoint of the anterior commissure–posterior commissure (AC–PC) line and 10–13 mm lateral and 4–6 mm below the AC–PC plane. Subsequently, the coordinate was adjusted under T2 imaging direct targeting. After the target and trajectory were planned, the DBS electrodes (Model 1210-40, SceneRay China or Model 3387S, Medtronic, USA) and the internal pulse generator (IPG, Model SR1101, SceneRay, or Model 37,612, Medtronic) were implanted under general anesthesia. CT scanning was performed immediately after lead implantation for all patients to exclude intracranial hemorrhage and fused to the preoperative MR images to confirm the electrode coordinates [24]. We performed staged surgery in the first two patients. After confirming the effect of 1-week temporary stimulation, the procedure of implanting the pulse generator was performed in the second stage.

### Deep brain stimulation programming

The same physician tested and optimized the DBS programming parameters for each patient during a 3-days period immediately after surgery. The initial parameters were screened from the lowest contacts with high-frequency monopolar stimulation; the goal was to achieve the greatest anticipated effect on tics while reducing the risk of adverse side effects [25]. Further DBS programming sessions and clinical outcome assessments, both conducted by a psychiatrist and a neurologist, took place as frequently as needed, or if requested by the patients, during visits to the outpatient clinic. The most effective contacts were located at the dorsal part of the STN, the same as reported by Martinez-Torres et al. [14]. The resulting DBS programming parameters at the 6-months follow-up are presented in Table 2.

**Table 2:** Stimulation parameters with best clinical responses

Patient	Electrode model	Left side				Right side			
		Amplitude /V	Pulse width /μs	Frequency /Hz	Contacts	Amplitude /V	Pulse width /μs	Frequency /Hz	Contacts
1	SceneRay 1210	4.5	130	120	C + 6–7-	4.5	120	160	C + 1–2-
2	SceneRay 1210	2.95	70	145	C + 6–7-	2.75	70	160	C + 2–3-
3	Medtronic 3387	3	70	160	C + 10–11-	3	90	160	C + 2–3-
4	Medtronic 3387	1.6	60	130	C + 10–11-	2.1	60	130	1 + 2-
5	SceneRay 1210	2.75	60	130	C + 6–7-	3.05	60	145	C + 2–3-
6	SceneRay 1210	2.45	90	125	5 + 6-	2.35	90	105	1 + 2-
7	SceneRay 1210	3.35	60	125	C + 6–7-	3	60	160	C + 2–3-
8	SceneRay 1210	2.9	90	135	6 + 7-	2	90	135	2 + 3-
9	SceneRay 1210	3	60	160	C + 6–7-	3.25	60	125	C + 2–3-
10	SceneRay 1210	4.25	70	145	C + 6–7-	3.05	50	105	C + 1–2-

C, Case; –, negative; + , positive. SceneRay lead configuration: right side: 0.1.2.3, left side: 4.5.6.7. Medtronic lead configuration: right side: 0.1.2.3, left side: 8.9.10.11

### Clinical outcome assessment

We focused on the following clinical outcomes: (1) severity of motor tics, severity of vocal tics, and overall impairment for motor and phonic tics, evaluated using the YGTSS; (2) severity of comorbid psychiatric symptoms, particularly symptoms of OCB, ADHD, anxiety, and depression, measured using the Yale–Brown Obsessive–Compulsive Scale (Y-BOCS), ADHD Rating Scale IV (ADHD-RS-IV), 14 items of the Hamilton Anxiety Rating Scale (HAMA-14), and 17 items of the Hamilton Rating Scale for Depression, respectively; and (3) psychological, social, and occupational functioning and quality of life, assessed using the Global Assessment of Functioning (GAF) and Gilles de la Tourette Syndrome-Quality of Life (GTS-QOL) rating scale. Clinical outcome data were obtained at the time of surgery (baseline), 3-months follow-up (3-mo FU), 6-month follow-up (6-mo FU), and 12-months follow-up (12-mo FU).

### Localization of deep brain stimulation electrodes

DBS electrode placement was reconstructed per patient using a state-of-the-art processing pipeline as implemented in Lead-DBS software version 2.5 [26] (https://lead-dbs.org). In brief, postoperative CT scans were linearly co-registered to preoperative MRI using Advanced Normalization Tools (http://stnava.github.io/ANTs/) [27]. This step was further enhanced through brain shift correction as included in Lead-DBS. Normalization into ICBM 2009b NLIN asymmetric (“MNI”) space was then performed for pre- and postoperative images using the symmetric diffeomorphic image registration (SyN) approach provided by ANTs [27], with the addition of a subcortical refinement step (“Effective: Low Variance + Subcortical Refinement” preset in Lead-DBS). Co-registration and normalization results were visually inspected and refined, where needed, with particular focus on the atlas fit at the level of the stereotactic target region. Automatic pre-localizations of electrodes were performed using the PaCER algorithm [28], manually refined by two expert users (BH and NL) and warped into MNI space.

### Estimation of the focal stimulation effect

Accounting for patient-specific DBS stimulation parameters, volumes of tissue activated (VTAs) were estimated using a finite element method [26]. A volume conductor model based on a four-compartment mesh covering gray and white matter, lead contacts, and insulating parts was applied. Simulation of the electric field distribution was achieved using the FieldTrip-SimBio pipeline integrated into Lead-DBS software (https://www.mrt.uni-jena.de/simbio/, http://fieldtriptoolbox.org/). A threshold at a heuristic value of 0.2 V/mm was applied to the stimulation effect to obtain binarized VTAs.

### Functional electrode connectivity computation

A profile of global functional electrode connectivity was modeled across all patients with TS included in the present study. For lack of patient-specific functional imaging data in the TS cohort itself, connectivity was approximated using a high-resolution connectome derived from resting-state functional MRI scans of 1000 healthy subjects [29], which had been acquired within the scope of the Brain Genomics Superstruct Project (https://dataverse.harvard.edu/dataverse/GSP) [30].

Seeding from bilateral VTAs, estimates of normative whole-brain functional connectivity were derived for each of the ten patients with TS using a similar strategy as employed in previous studies [31, 32]: On a voxel-by-voxel basis, averaged time series of voxels inside each bilateral VTA were correlated with all remaining voxels in the whole brain. Per patient with GTS, this procedure was repeated for each of the 1000 subjects in the normative sample, and results were subsequently averaged and Fisher-*z*-transformed. Since VTA seeds differed across patients with TS in dependence of individual electrode placement and stimulation parameter settings, each patient received one average functional connectivity *fingerprint* following this approach. Averaging across the fingerprints of all ten patients in the GTS sample finally yielded one global profile of functional electrode connectivity. Methods applied to derive anatomical regions corresponding to peaks within this functional connectivity profile are reported in the supplementary materials.

### Statistical analyses

Initially, data inspection showed a non-normal distribution. Accordingly, these data were submitted to a nonparametric approach, using the Friedman and Dunn tests for post-hoc pairwise comparisons. These tests were used to analyze the data, including time as a within-subject factor with four levels (baseline, 3-mo FU, 6-mo FU, and 12-mo FU). To explore possible differential treatment effects on motor and vocal tics, a Wilcoxon matched pairs test was used to determine whether improvements in motor and phonic tics attained at the last follow-up differed from each other in magnitude. Quantitative variables are presented as mean ± standard deviation and ranges; qualitative variables are presented as counts and percentages. A *p* value of < 0.05 was considered significant for overall tests; the *p* values for the Dunn test were adjusted for multiple comparisons. GraphPad Prism 8 software (GraphPad Software, Inc., San Diego, CA) was used for statistical analyses and plotting.

## Results

### Patient characteristics

Ten patients (nine male and one female; age of onset, 8.3 ± 2.9 [range 5–12] years) were enrolled in this study. At time of surgery, the mean age of the patients was 23.1 ± 9.4 (range 14–38) years. The medical history indicated that patients were refractory to commonly prescribed medication, including *α*2 agonists (e.g., clonidine), antipsychotics (e.g., haloperidol and aripiprazole), and dopamine antagonists (e.g., tiapride). All patients experienced severe and refractory TS, resulting in significant functional impairment, distress, and a poor quality of life. The demographic and clinical characteristics of each patient are summarized in Table 1.

### Tic severity

**Fig. 1:**
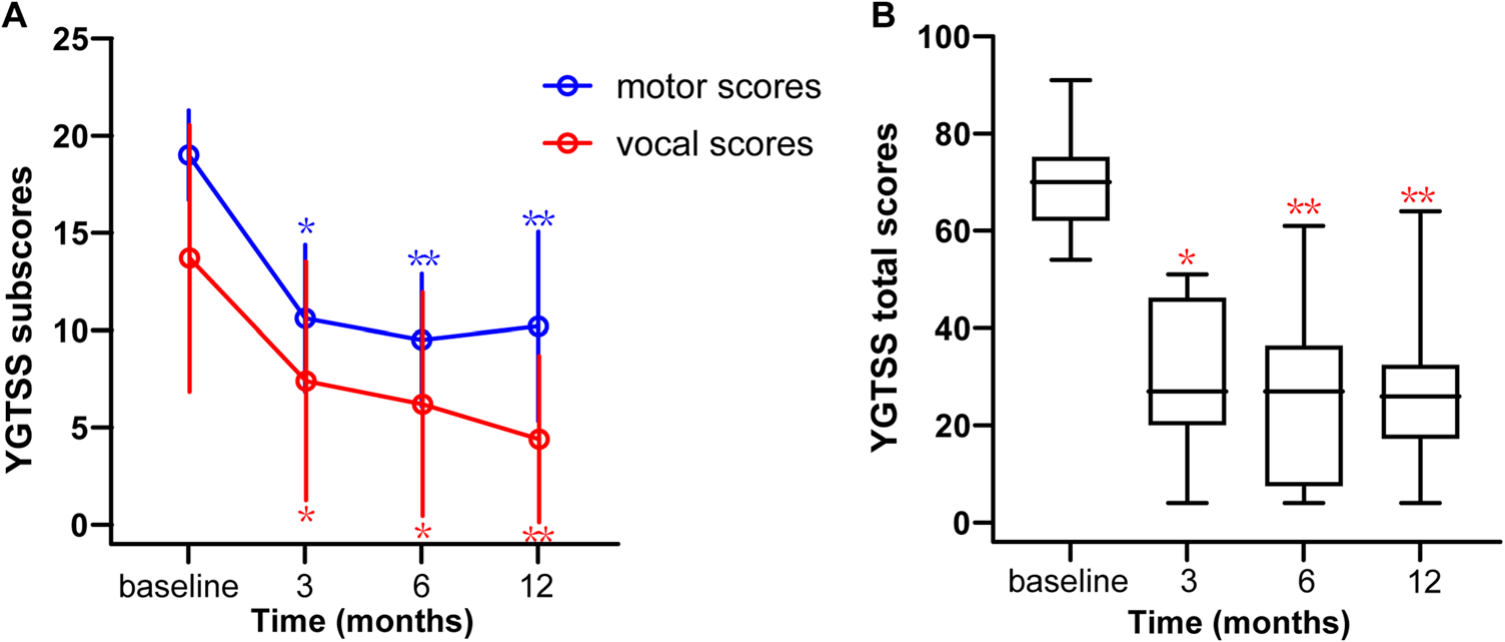
Tic severity over time. **a** Line chart of motor and vocal tics and **b** box plot of overall tic severity evaluated based on the YGTSS total scores at baseline and at the 3-, 6-, and 12-month follow-ups. Error bars indicate standard deviations of the scores. Note: ^*^p < 0.05. ^**^p < 0.01 (relative to baseline). Abbreviation: *YGTSS* Yale Global Tic Severity Scale

Relative to the severity of their tics at baseline, overall GTS severity, as indexed by the YGTSS total scores, showed clinically meaningful reduction after surgery, with 55.7% ± 24.5% (*p* = 0.0194), 62.9% ± 26.2% (*p* = 0.0004), and 58.8% ± 27.2% (*p* = 0.0032) tic improvement at 3-mo FU, 6-mo FU, and 12-mo FU, respectively. The severities of both motor and vocal tics and associated impairments, as measured based on the YGTSS subscale scores, were significantly reduced after surgery. At the 6-mo FU, vocal and motor tics were significantly improved by 64.5% ± 33.0% and 55.2% ± 34.7%, respectively, relative to baseline. The improvement of vocal tics was up to 74.8% ± 24.3% and the motor tics (46.6% ± 23.1%) trended to stable at the 12-mo FU period (Table 3, Fig. 1).

**Table 3:** Clinical assessment of outcomes and post-hoc analyses

Outcome variable	Baseline	3-mo FU	6-mo FU	12-mo FU	Friedman test *p* value	Adjusted *p* value		
						Baseline vs. 3-mo FU	Baseline vs. 6-mo FU	Baseline vs. 12-mo FU
YGTSS-motor	19.0 (2.3)	10.6 (3.8)	9.5 (3.4)	10.2 (4.9)	0.0001	0.0256	0.0008	0.0012
YGTSS-vocal	13.7 (6.8)	7.4 (6.1)	6.2 (5.8)	4.4 (4.3)	< 0.0001	0.0436	0.0109	< 0.0001
YGTSS-impairment	37.0 (10.6)	12.0 (7.9)	10.0 (11.6)	13.0 (12.5)	0.0039	0.0919	0.0109	0.0919
YGTSS-total	69.7 (10.2)	30.0 (15.1)	25.7 (17.7)	27.6 (16.9)	0.0002	0.0194	0.0004	0.0032
Y-BOCS	3.5 (6.5)	1.2 (2.6)	2.5 (4.6)	0.8 (2.5)	0.4160	> 0.9999	> 0.9999	> 0.9999
HAMD-17	5.1 (5.5)	1.1 (1.4)	2.1 (4.7)	2.8 (3.6)	0.0137	0.1162	0.0919	> 0.9999
HAMA-14	6.2 (5.0)	2.2 (2.6)	2.2 (3.6)	4.2 (4.3)	0.0034	0.2783	0.0194	> 0.9999
ADHD-RS-IV	28.0 (6.9)	24.1 (5.6)	23.8 (6.9)	26.0 (7.1)	0.0803	0.2260	0.2260	> 0.9999
GTS-QOL	24.3 (9.7)	14.2 (8.4)	15.2 (13.9)	13.2 (8.8)	0.0191	0.1461	0.0919	0.0562
GAF	65.4 (7.4)	75.7 (5.5)	79.9 (6.9)	79.6 (1.7)	0.0003	0.2260	0.0004	0.0060

Data are presented as means with standard deviations in parentheses

Abbreviations: *3-mo FU* 3-month follow-up, *6-mo FU* 6-month follow-up, *12-mo FU* 12-month follow-up, *ADHD-RS-IV* Attention-Deficit/Hyperactivity Disorder Rating Scale IV, *GAF* Global Assessment of Functioning Scale, *GTS-QOL* Gilles de la Tourette Syndrome-Quality of Life Scale, *HAMA-14* 14 items of the Hamilton Anxiety Scale, *HAMD-17* 17 items of the Hamilton Depression Scale, *Y-BOCS* Yale-Brown Obsessive Compulsive Scale, *YGTSS* Yale Global Tic Severity Scale

### Psychiatric comorbidity severity

**Fig. 2:**
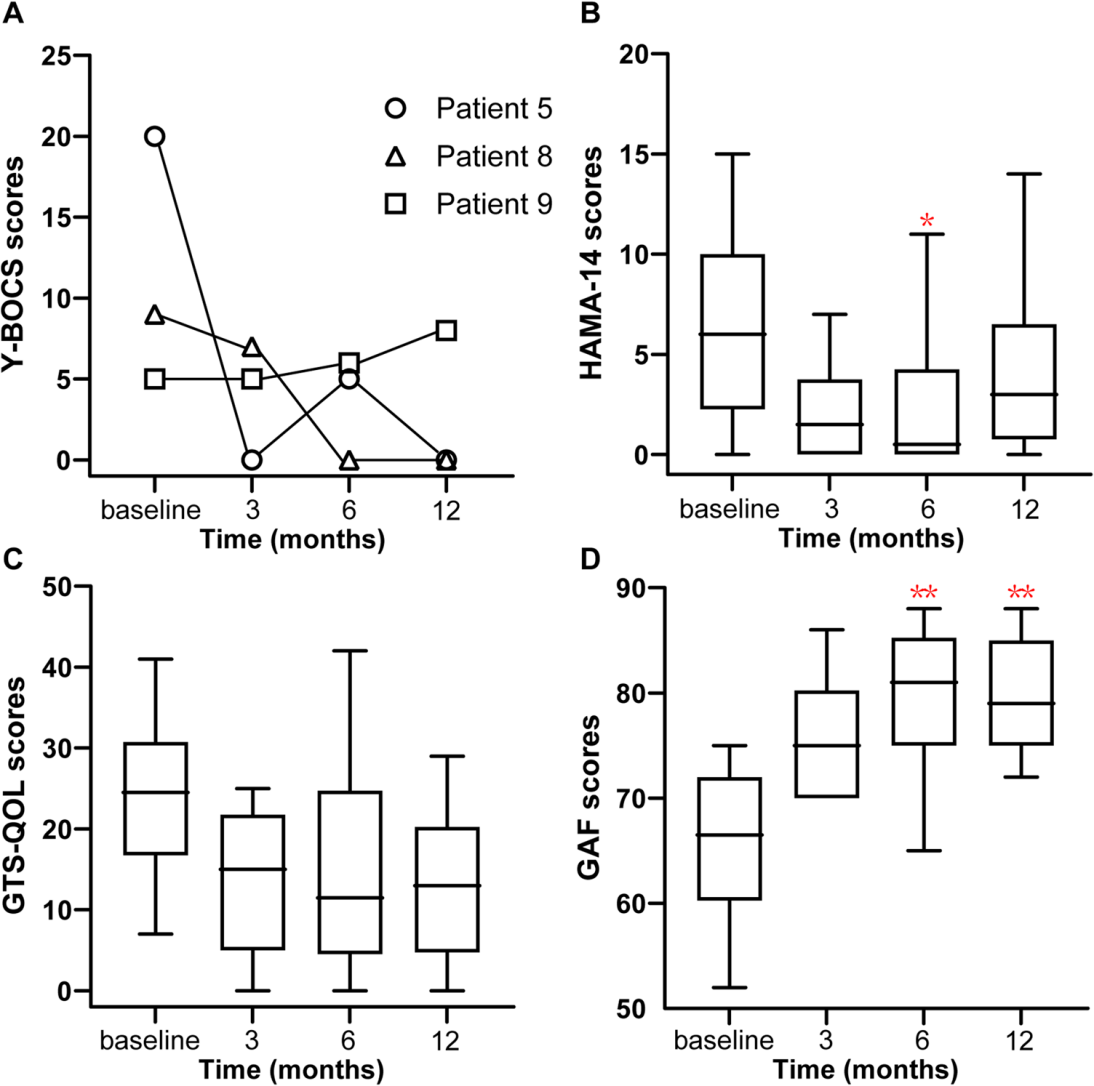
Psychiatric comorbidities and quality of life over time. **a** Line chart of OCB evaluated using the Y-BOCS. **b**–**d** Box plot of anxiety, quality of life, and adaptive functioning evaluated using the HAMA-14, GTS-QOL, and GAF, respectively. Note: ^*^p < 0.05; ^**^p < 0.01 (relative to baseline). Abbreviations: *GAF* Global Assessment of Functioning scale, *GTS-QOL* Gilles de la Tourette Syndrome-Quality of Life Scale, *HAMA-14* 14 items of the Hamilton Anxiety Scale, *Y-BOCS* Yale-Brown Obsessive Compulsive Scale

Among the patients included in this study, three had comorbid OCD (patients 5, 8, and 9). The severity of OCD symptoms, as measured using the Y-BOCS, improved by 51.7% at the 6-mo FU period. Patient 5 experienced moderate depression before surgery and a significant improvement after STN-DBS. On a group level, HAMD and ADHD-RS-IV showed no significant reductions after surgery. HAMA-14 scores significantly reduced at the 6-mo FU period (Fig. 2B), with a symptom improvement of 65.1% (*p* = 0.0417) across the entire cohort, but with no significant difference at 12-mo FU period (Table 3).

### Quality of life

Importantly, patients’ social, occupational, and academic functioning had been profoundly impaired due to their severe GTS and psychiatric comorbidities before surgery. Quality of life, measured based on the GTS-QOL score, improved by 39.0 ± 49.6% and 35.4 ± 64.2% at the 6-mo and 12-mo FU periods, respectively (Fig. 2C). Adaptive functioning, as indexed by the GAF, increased significantly at the 6-mo FU (*p* = 0.0004) and 12-mo FU (*p* = 0.006) periods, with an average improvement of 22.8% ± 10.5% and 23.4% ± 18.5%, respectively (Table 3, Fig. 2D).

### Complications and side effects

Patients did not experience significant complications or serious side effects associated with surgery or hardware. The most commonly reported side effects at the perioperative period were light headache, dizziness, and fatigue, which lasted for a short period of time and did not affect daily activities. Eight patients experienced a short or long period of light dysarthria during the course of the treatment, which seemed to be related to DBS. In addition, adverse side effects related to subthalamic stimulation were mostly temporary and included dysarthria (*n* = 1), light salivation (*n* = 2), thirst (*n* = 1), and sleepiness (*n* = 1). All these adverse effects could be alleviated by means of DBS parameter adjustments. Patient 5 reported noticeable weight gain after surgery (60–77.5 kg in a period of 6 months). Following a period of symptom relief, patient 4 experienced a recurrence of tics at the 6-mo FU period, and symptoms of OCB, anxiety, and depression were also observed. Symptoms improved after adjustment of the parameters, which required a subsequent follow-up visit and observation. Two patients (patients 6 and 7) who forgot to recharge the IPG experienced an unwanted, accidental stimulation discontinuation of DBS for a week before the 6-mo FU period. Surprisingly, tic symptoms did not relapse severely during these periods of 1 week off DBS.

### Profile of functional electrode connectivity

**Fig. 3:**
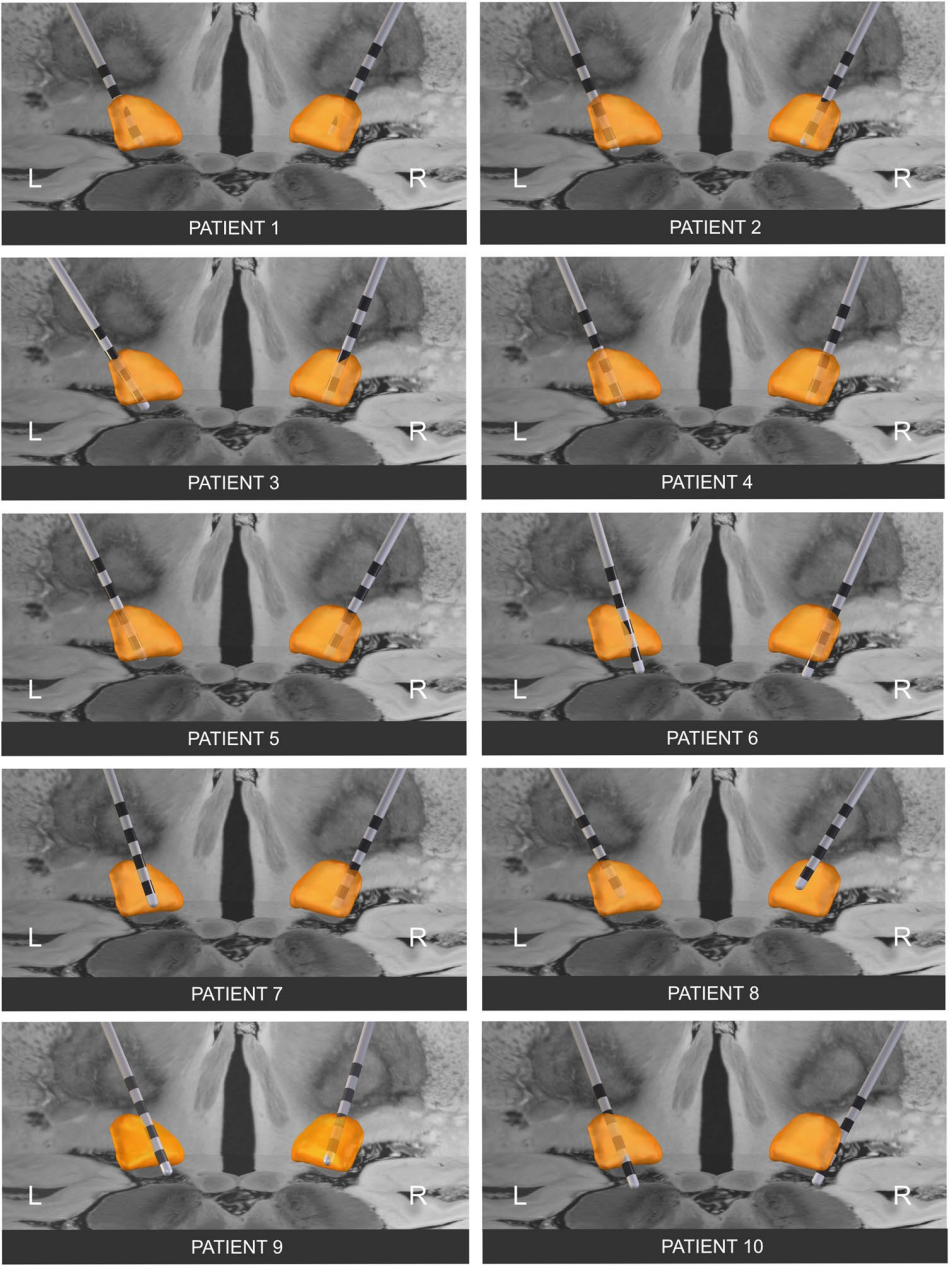
Reconstruction of DBS electrode placement using Lead-DBS software. All the active contacts reached the dorsal part of the subthalamic nucleus. Abbreviations: *DBS* deep brain stimulation

**Fig. 4:**
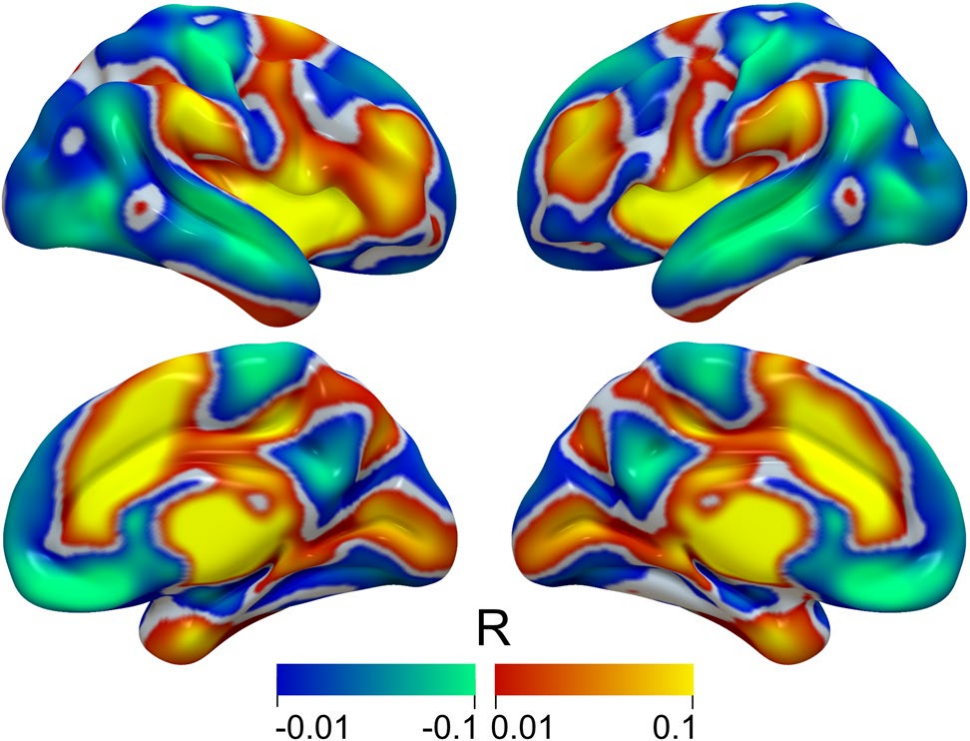
The average profile of functional electrode connectivity in multiple whole-brain regions. Positive correlations are presented in warm colors, and negative correlations are shown in cool colors

A whole-brain map of functional connectivity related to the stimulation effect was derived based on the precise electrode location of ten patient-wise bilateral pairs of active electrodes (i.e., 20 electrodes in total). Electrode reconstruction confirmed accurate placement of DBS leads within the dorsal aspect of the STN for all patients (Fig. 3). The average profile of functional electrode connectivity indicated a set of voxels associated with the focal stimulation effect produced by active electrodes in multiple whole-brain regions (Fig. 4). Anatomical correlates of peaks within this map included the thalamus, pallidum, substantia nigra pars reticulata, putamen, insula, and mid- and anterior cingulate cortices. Brain sites associated with anticorrelated peaks comprised middle temporal, rectal, and superior temporal gyri. A more comprehensive overview of regions that were, on average, positively (Table S1) or negatively (Table S2) functionally related to the local stimulation effect can be found in the supplemental materials.

## Discussion

Our study showed significant improvements in both motor and vocal tics following1 year of STN-DBS. We observed a relatively fast effect of STN-DBS on GTS symptoms. A study including data on a subset of patients from the International TS DBS Database and Registry in coordination with the International Neuromodulation Registry at the University of Utah found that the median periods of response (≥ 40% reduction in YGTSS total score) for patients targeting the anteromedial GPi and centromedian nucleus of the thalamus were 18 and 11 months, respectively [6]. A systematic review and meta-analysis revealed 48% improvement after thalamic stimulation and 55–58% improvement after GPi stimulation at the last available follow-up [4]. In our study, the YGTSS total scores improved by an average of more than 60% at the 6-mo FU period. Although a direct head-to-head comparison in the form of a blinded clinical trial is lacking, the potentially more rapid clinical response of STN-DBS compared to GPi or centromedian DBS could turn out as an advantage of this novel target for GTS. In this study, the patients’ quality of life and adaptive functioning improved significantly at the 12-mo FU period, but overall anxiety scores showed no significant difference relative to baseline. According to the patients, the recurrence of anxiety was related to the stress experienced in their environment after they returned to work and school.

GTS is pathophysiologically associated with aberrant neural activity that disrupts normal cortico-striato-thalamo-cortical (CSTC) circuit function [33]. Regarding the CSTC circuits, the STN occupies a privileged position connecting specific regions in the frontal cortex to the basal ganglia and thalamus, which regulates complex behaviors [1]. According to several studies, the STN is associated with behavioral changes, which may be improved by STN-DBS [34–36]. The most positive outcomes of relieving tic symptoms in our cohort of patients were observed as active contacts mostly located at the dorsal part of the STN. The dorsal STN, also referred to as the sensorimotor subregion, is positioned closed to the zona incerta, Forel’s field H [37, 38], and cerebello- and pallidothalamic projections [39] that are associated with motor regulation. Through the analysis of DBS electrode placement and normative connectome mapping [40], functional connectivity between DBS stimulation sites within the surgical target structure with the motor cortex and pre- and supplementary motor areas emerged [41]. These connections can represent hyperdirect input from cortical sites to the STN. Moreover, the sensorimotor functional zone of the nucleus is located at its dorsolateral side, matching its therapeutic target for movement disorders. According to our whole-brain map, stimulation effect of the sensorimotor STN showed correlated functional connectivity to the thalamus, pallidum, substantia nigra pars reticulata, and putamen. Higher tic severity was associated with enhanced activation of the substantia nigra and striatal and thalamic regions in the direct pathway [42], which suggests that subthalamic stimulation can regulate these brain sites to modulate the onset of tic symptoms.

Certainly, the small size of the STN may more easily regulate abnormal neuronal activity in the limbic and sensorimotor subregions compared to the GPi of thalamic DBS [14]. DBS targeting the limbic STN has been effective for OCB [18]. Li et al. reported that connectivity to the anterior cingulate cortex (ACC) and insula was predictive, regardless of the DBS targeting the anterior limb of the internal capsule or STN zones for OCB [43], as these brain regions are also the correlated peak within the map in our study. The dorsal ACC hyperactivity in patients with OCB is relative to that in controls, suggesting that it supposedly plays a prominent role in OCB pathophysiology [44]. The efficacy of dorsal cingulotomy has been confirmed in patients with treatment-resistant OCB [45]. Above all, the dorsal site of STN-DBS can improve not only tic symptoms by targeting the sensorimotor subregion but also OCB symptoms potentially by influencing the associative-limbic subregion.

STN-DBS has a positive effect on motor symptoms of movement disorders; however, according to previous studies, STN-DBS might cause speech and voice deterioration [36, 46, 47]. In this study, vocal tics showed significant improvement in our patients, but some patients experienced light dysarthria as a consequence of high-frequency stimulation. High-frequency stimulation may result in a possible spreading of the electric current to the corticobulbar and corticospinal tracts [48], leading to a negative stimulation only in the fibers involved in the motor control of speech and a positive effect in the fibers involved in phonation [49, 50]. Taken together, these findings may be indicative of the importance to achieve a balance between motor control and speech impairments in these patients.

The STN-DBS treatment was reasonably well tolerated by the patients with GTS, but various adverse events were documented after surgery. In one patient, significant weight gain was observed 6 months after surgery. In some patients with PD and dystonia undergoing STN-DBS, this was also observed [51–53]. Certainly, previous studies have suggested a change in body weight as a result of the regulation of diet and metabolism by STN-DBS through diffusion of the electrical pulse to the hypothalamus [54, 55]. Two patients who failed to recharge the IPG experienced an unwanted, accidental stimulation discontinuation of DBS for a week around the time of the 5–6-months follow-up. Surprisingly, their tic symptoms did not relapse severely during this brief discontinuation period. This is consistent with the experience of several other studies in GTS [56–58]. The normalization of cortical or subcortical plasticity, induced by long-term stimulation, may account for the maintenance of the achieved clinical benefits [59, 60]. However, this phenomenon was observed after a short-term stimulation in our study, suggesting that the correction of plasticity may occur significantly earlier than previously assumed.

Although our observations should be regarded as preliminary, the results reported herein provide the first evidence indicating that STN-DBS may be an effective and safe option for managing tics in patients who experience severe and refractory GTS. The results of this observational study, as those of the majority reported in the GTS DBS literature at present, are limited by the small sample size and lack of randomization, blinding, and a control group. Compared to the one of patients included in other trials, the age of the patients in the present study was relatively young. Although no age limit for DBS trials for GTS has been suggested by the Tourette Association of America in 2015 [2], its use in younger patients remains limited. Thus, it is essential for a multidisciplinary team to discuss whether DBS is suitable in younger patients.

## Supplementary Information

Below is the link to the electronic supplementary material.

Supplementary file1 (DOCX 28 KB)
