# Correction: Subthalamic deep brain stimulation for refractory Gilles de la Tourette’s syndrome: clinical outcome and functional connectivity

**DOI:** 10.1007/s00415-022-11327-0

**Published:** 2022-08-18

**Authors:** Lulin Dai, Wenying Xu, Yunhai Song, Peng Huang, Ningfei Li, Barbara Hollunder, Andreas Horn, Yiwen Wu, Chencheng Zhang, Bomin Sun, Dianyou Li

**Affiliations:** 1 Department of Neurosurgery, Center for Functional Neurosurgery, Ruijin Hospital, Shanghai Jiao Tong University School of Medicine, Shanghai, China; 2 Department of Neurosurgery, Shanghai Children’s Medical Center, Affiliated to the Medical School of Shanghai Jiao Tong University, Shanghai, China; 3 Movement Disorders and Neuromodulation Unit, Department of Neurology, Charité - Universitätsmedizin Berlin, Berlin, Germany; 4 Einstein Center for Neurosciences Berlin, Charité - Universitätsmedizin Berlin, Berlin, Germany; 5 Berlin School of Mind and Brain, Humboldt-Universität zu Berlin, Berlin, Germany; 6 Center for Brain Circuit Therapeutics, Department of Neurology, Brigham and Women’s Hospital, Boston, MA USA; 7 MGH Neurosurgery and Center for Neurotechnology and Neurorecovery (CNTR) at MGH Neurology, Massachusetts General Hospital, Boston, MA USA; 8 Department of Neurology, Center for Functional Neurosurgery, Ruijin Hospital, Shanghai Jiao Tong University School of Medicine, Shanghai, China; 9 Shanghai Research Center for Brain Science and Brain-Inspired Technology, Shanghai, China

## Correction: Journal of Neurology 10.1007/s00415-022-11266-w

The original version of this article unfortunately contained a mistake. In figure 3, the electrode localizations of all patients do not match the one from the table.

The corrected Fig. 3 is given in the following page.

**Fig. 3:**
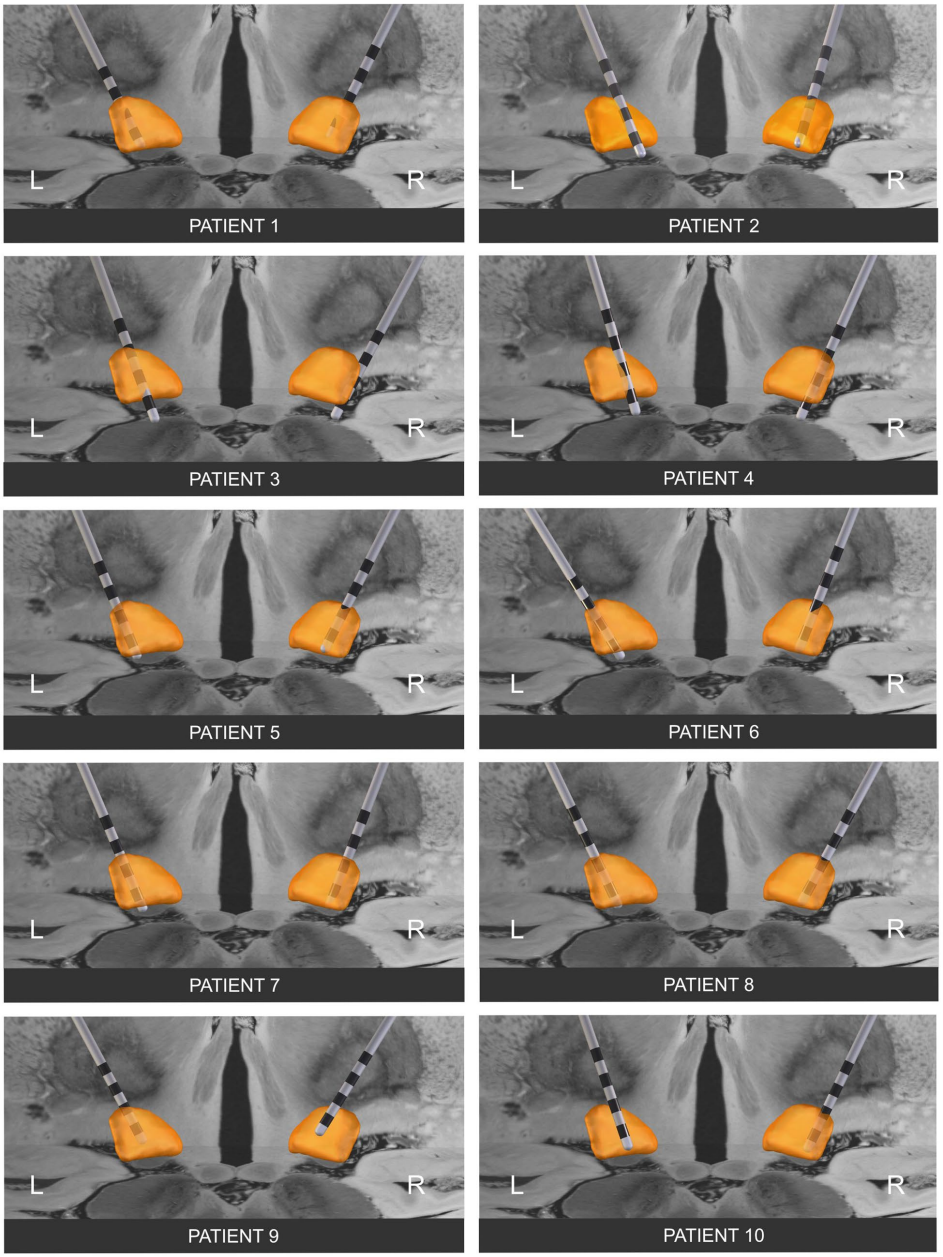
Reconstruction of DBS electrode placement using Lead-DBS software. All the active contacts reached the dorsal part of the subthalamic nucleus. Abbreviations: *DBS* deep brain stimulation

