# Supplementary material for: Subthalamic deep brain stimulation for refractory Gilles de la Tourette’s syndrome: clinical outcome and functional connectivity
Source: J Neurol. 2022 Jul 21;269(11):6116–26. doi: 10.1007/s00415-022-11266-w (PMC9553760; doi:10.1007/s00415-022-11266-w)
Supplement: Supplementary file 1 — Supplementary file1 (DOCX 28 KB) [file 415_2022_11266_MOESM1_ESM.docx]

**Supplementary Information**

**﻿**

1. **Prominent anatomical regions**

Peak Z-values as well as corresponding MNI-coordinates were extracted for the normative group-level functional connectivity map that had been averaged across fingerprints seeding from each patient’s volume of tissue activated. For this purpose, peak clusters and local maxima were anatomically labeled based on probabilistic cytoarchitectonic maps via the SPM Anatomy Toolbox Version 2.2b[1, 2]. Subsequently, results were visually inspected and manually refined where needed.

**﻿Table S1 Anatomical regions positively associated with the focal stimulation effect**

| **MNI-Coordinates (in mm)** | | | | | **Averaged Z-Value** |
| --- | --- | --- | --- | --- | --- |
| **Region** | **Hemisphere** | **X** | **Y** | **Z** |  |
| Thalamus | LH | -14 | -12 | -2 | 0.92 |
|  | RH | 14 | -12 | 0 | 0.79 |
| Pallidum | LH | -22 | -6 | 0 | 0.35 |
|  | RH | -22 | 0 | 0 | 0.31 |
| Substantia Nigra, pars reticulata | LH | -12 | -12 | -10 | 0.35 |
|  | RH | 12 | -12 | -10 | 0.37 |
| Putamen | LH | -24 | -8 | 4 | 0.30 |
|  | RH | 30 | -14 | -2 | 0.28 |
| Insula | LH | -34 | 8 | 0 | 0.25 |
|  | RH | 38 | 10 | -4 | 0.23 |
| Midcingulate Cortex | LH | 2 | 20 | 34 | 0.21 |
|  | RH | 4 | 22 | 32 | 0.21 |
| Anterior Cingulate Cortex | LH | -4 | 20 | 30 | 0.20 |
|  | RH | 4 | 24 | 28 | 0.20 |
| Supramarginal Gyrus | RH | 60 | -32 | 34 | 0.13 |
|  | LH | -60 | -36 | 30 | 0.11 |
| Cerebellum (VIII) | LH | -36 | -50 | -52 | 0.13 |
|  | RH | 36 | -46 | -52 | 0.12 |
| Middle Frontal Gyrus | LH | -30 | 38 | 30 | 0.11 |
|  | RH | 32 | 42 | 24 | 0.13 |
| Precuneus | LH | -18 | -44 | 2 | 0.11 |
|  | RH | 22 | -42 | 2 | 0.12 |

**﻿**

**Table S2 Anatomical regions negatively associated with the focal stimulation effect**

| **MNI-Coordinates (in mm)** | | | | | **Averaged Z-Value** |
| --- | --- | --- | --- | --- | --- |
| **Region** | **Hemisphere** | **X** | **Y** | **Z** |  |
| Middle Temporal Gyrus | LH | -64 | -22 | -4 | -0.14 |
|  | RH | 64 | -26 | -2 | -0.14 |
| Rectal Gyrus | LH | 0 | 34 | -16 | -0.14 |
|  | RH | 2 | 36 | -16 | -0.14 |
| Superior Temporal Gyrus | LH | -56 | -6 | -10 | -0.12 |
|  | RH | 64 | -24 | 0 | -0.15 |
| Angular Gyrus | LH | -54 | -64 | 34 | -0.12 |
|  | RH | 58 | -58 | 26 | -0.10 |
| Postcentral Gyrus | LH | -42 | -34 | 66 | -0.13 |
|  | RH | 42 | -28 | 66 | -0.13 |
| Inferior Temporal Gyrus | LH | -66 | -22 | -18 | -0.09 |
|  | RH | 52 | -72 | -4 | -0.14 |
| Middle Occipital Gyrus | LH | -50 | -78 | 4 | -0.12 |
|  | RH | 54 | -76 | -2 | -0.12 |
| Primary Motor Area (Area 4a) | - | 0 | -32 | 62 | -0.12 |
| Posterior Cingulate Cortex | LH | -2 | -52 | 32 | -0.11 |
|  | RH | 2 | -54 | 30 | -0.11 |

**References**

1. Eickhoff SB, Stephan KE, Mohlberg H, et al (2005) A new SPM toolbox for combining probabilistic cytoarchitectonic maps and functional imaging data. Neuroimage 25:1325-35. <http://doi.org/10.1016/j.neuroimage.2004.12.034>

2. Eickhoff SB, Paus T, Caspers S, et al (2007) Assignment of functional activations to probabilistic cytoarchitectonic areas revisited. Neuroimage 36:511-21. <http://doi.org/10.1016/j.neuroimage.2007.03.060>
